# Meeting of the State Medical Society

**Published:** 1857-08

**Authors:** 


### EDITORIAL AND MISCELLANY.

### Meeting of the State Medical Society.

We publish the proceedings of our State Medical Society in the present number. It will be seen that there was a decided disposition on the part of our brethren of Iowa City, and the northern part of the state, to censure the officers and committees, of last year, as also the members of the society from the southern portion of the state, for non-attendance &c.

We cordially coincide in opinion with the members in attendance, that there should be a greater degree of interest felt in the meetings of the society, and a greater effort put forth to make the meetings what they ought to be, in order to secure a full attendance from year to year. But it is a question whether the indiscriminate censure of our friends, at the last meeting, is in entire good taste when we remember that the great majority of those present were so for the first time, and that of the remainder, only a very few were ever in attendance upon more than one other meeting, while a number of those whom they condemned, have, from the first formation of the society, made it a rule to be present at all its meetings.

On behalf of the publishing committee, D. C. Dewey, M. D., chairman, we are requested to state that the proceedings of last year's meeting at Ottumwa, and also a synopsis of the years 1854–5, were published, as directed by the society, but by some miscarriage in their arrangements with the Treasurer, the expense of publishing was not paid. The chairman of that committee being absent from the state at the time, Dr. M'Gugin, another of the committee and also chairman of one of the standing committees of the society, had made arrangements to attend the meeting in Iowa City, and report for both committees. Circumstances beyond his control, (such as every medical man knows will at times arise, peremptorily preventing absence,) occurred just at a time when, if he would be present at the meeting, he must have started, and delayed until too late to go. The consequence was that it was impossible at that time to send on the published proceedings so as to have them arrive until after the meeting should have adjourned.

Thus it will be seen that the delinquency of the publishing committee was one of necessity, and entirely excusable, since it was unforeseen and would have been unprovided for even by the most provident of committees.

The same excuse will of course apply to the report of the committtee on Pathology and Practice, of which Dr. M'Gugin was chairman. So much we have stated by request. We are glad if a disposition may be aroused in the profession in the state, to hold themselves responsible for the prompt discharge of duty to the society. But there should ever be great caution in public censure of individual members, or of general censure.

It is an easy matter to see faults in others, but it is not always easy for ourselves to discharge, to the full, our duties. It is ever but common charity to suppose there is sufficient excuse for apparent dereliction until a fair opportunity is offered for the delinquent to explain himself, and he shall then have an opportunity of clearing himself of the imputed sin.

We confess we are sorry the resolutions were passed as they were until the society had taken the proper steps to ascertain if so severe a censure were really deserved. It had been better in our opinion (quite likely we are mistaken, for we claim no such thing as infallibility,) to have made it the duty of a special committee to inquire into the matter and report to another meeting, when action could be taken and *undoubted* justice be administered.

We have no disposition to consider the action of the society as intentionally wrong, nor do we believe the profession throughout the state will impute wrong motives to the movers in such a cause.

The next meeting will be held at Mt. Pleasant on the 2d Wednesday in June next. Let the profession of the state busy themselves from this time forth with preparations to make that meeting what it should be. We hope every committee will be prompt to make full reports upon subjects referred to them, and that individuals will take it upon themselves to prepare reports upon whatever they may think will be of interest to the society.

Let the next annual meeting have a general attendance from the whole state, and all local, or other ill feeling, will fade out under the genial influence of social intercourse and the interchange of professional and gentlemanly courtesy.

W. R. M.

Since the above was written I have been requested by Dr. J. C. Hughes, late President of the society, (now absent from the city,) to state that, upon finding it would be impossible for him to attend the meeting at Iowa City—because of absence, unexpectedly protracted to several weeks, in the east, just previous to the meeting—he wrote to the vice president, stating the fact, and asking him, if possible, to be present and preside.

Dr. H. did not learn anything of the vice president's inability to attend the meeting until the occasion had passed by. when it was of course too late to do anything in the matter,

We give herewith extracts from a letter received from Prof. M'Gugin:—

KEOKUK, July 20, 1857.

*Drs. Hughes and Marsh*:—It pained me much to observe in the report of the proceedings of the State Medical Society, that expressions denunciatory to certain absent officers and members of the committees were indulged in.

While I rejoice to perceive a disposition on the part of the membership to demand of every one, charged with the performance of a duty, an implicit compliance with the requirements, yet I am constrained in self-defence to say that, in the instance above alluded to there was a manifest want of liberality, and that judgment was rendered without a trial.

Dr. Cochran, who was present, well knows from a letter received from me, that it was my intention to be present, for *I told him so.* The Dr. could have said so, had he *remembered* the fact.

I left my practice and attended meetings at Davenport and Muscatine, when the intelligent faces of the movers of the resolutions of censure were not seen. * * * *

The only members of the State Medical Society, who visited Nashville, at the late meeting of the American Medical Association, were Dr. Hughes and myself—the former representing the society and myself the college—and this was done at much sacrifice of time, and some money.

With regard to the publishing committee, it would be well to state, that the whole duties devolved upon Dr. Dewey, who discharged them faithfully and well, whilst I was confined by a long, protracted and painful illness, during last fall. The Reports are here, subject to orders from the members, but as they cannot be sent out without prepayment, he did not feel himself justified in incurring such a heavy expense.

